# Fabrication Method for Shape-Controlled 3D Tissue Using High-Porosity Porous Structure

**DOI:** 10.3390/bioengineering11020160

**Published:** 2024-02-05

**Authors:** Hidetaka Ueno, Shohei Yamamura

**Affiliations:** 1 Center for Advanced Medical Engineering Research & Development (CAMED), Kobe University, 1-5-1 Minatojima-minamimachi, Chuo-ku, Kobe-city 650-0047, Hyogo, Japan; 2 Department of Medical Device Engineering, Graduate School of Medicine, Kobe University, 7-5-1 Kusunoki-cho, Chuo-ku, Kobe-city 650-0017, Hyogo, Japan; 3 Health and Medical Research Institute, National Institute of Advanced Industrial Science and Technology (AIST), 2217-14 Hayashi-cho, Takamatsu-city 761-0395, Kagawa, Japan

**Keywords:** high-porosity porous structure, micromesh, SU-8, 3D tissue, tissue engineering

## Abstract

Shape-controlled 3D tissues resemble natural living tissues in human and animal bodies and are essential materials for developing and improving technologies in regenerative medicine, drug discovery, and biological robotics. In previous studies, shape-controlled 3D tissues were fabricated using scaffold structures or 3D bioprinting techniques. However, controlling the shape of 3D tissues without leaving non-natural materials inside the 3D tissue and efficiently fabricating them remains challenging. In this paper, we propose a novel method for fabricating shape-controlled 3D tissues free of non-natural materials using a flexible high-porosity porous structure (HPPS). The HPPS consisted of a micromesh with pore sizes of 14.87 ± 1.83 μm, lattice widths of 2.24 ± 0.10 μm, thicknesses of 9.96 ± 0.92 μm, porosity of 69.06 ± 3.30%, and an I-shaped microchamber of depth 555.26 ± 11.17 μm. U-87 human glioma cells were cultured in an I-shaped HPPS microchamber for 48 h. After cultivation, the 3D tissue was released within a few seconds while maintaining its I-shape. Specific chemicals, such as proteolytic enzymes, were not used. Moreover, the viability of the released cells composed of shape-controlled 3D tissues free of non-natural materials was above 90%. Therefore, the proposed fabrication method is recommended for shape-controlled 3D tissues free of non-natural materials without applying significant stresses to the cells.

## 1. Introduction

The biological function of 3D tissues more closely resembles that of natural living tissues than that of 2D cell layers [1,2,3,4,5]. These 3D tissues are used in various types of research, such as regenerative medicine [6,7], drug discovery [8,9,10], and biological robotics [11,12,13]. Three-dimensional tissue should not contain non-natural materials (e.g., polymer compounds and hydrogels) to achieve a biological function closer to living natural tissue. Additionally, its shape should be controlled to be suitable for each research purpose in order to improve these technologies. However, the efficient fabrication of shape-controlled 3D tissues without non-natural materials remains challenging because biomaterials (natural materials) are soft and unstable.

There are two methods for fabricating 3D tissues: one involves adhering cells to a non-natural scaffold structure (e.g., polystyrene porous scaffolds) to control the shape of the tissue and the other involves controlling the shape of tissues using non-adhesive coating materials (e.g., aqueous solution of poly(ethylene oxide)-poly(propylene oxide)-poly(ethylene oxide) triblock copolymer) or the interface of liquid droplets [14,15,16]. In the former method, microstructures fabricated using techniques such as track etching [17,18], phase separation [19,20], electrospinning [21,22,23], and hydrogels [24,25,26] are utilized as non-natural scaffold structures (e.g., polystyrene porous membranes, polymer nanofibers, alginate) to support 3D tissues. These scaffold structures have microscale resolutions, allowing for the design of scaffold shapes according to the desired shape of the 3D tissue, thereby enabling control of the shape of the 3D tissue. However, because these 3D tissues have non-natural materials, the biological function of 3D tissue is not the same as that of natural 3D tissue, and their use in regenerative medicine is hindered because it is difficult to obtain approval for the insertion of non-natural materials inside the human body. However, techniques utilizing interfaces where cells cannot adhere, such as non-adhesive coating materials (e.g., aqueous solutions of poly(ethylene oxide)-poly(propylene oxide)-poly(ethylene oxide) triblock copolymer) or liquid droplet interfaces, make it possible to create 3D tissues free of non-natural materials [27,28,29,30]. When cells are spread into microchambers coated with cell-non-adhesive materials, they form spheroids without adhering to the surface of the microchamber. In the hanging drop method, the substrate is flipped to ensure that cells gather in the hanging droplet and are cultured in medium droplets. As the surface of the droplets is in contact with air, cells can form spheroids [31,32]. However, in culture systems utilizing non-adhesive coatings or the hanging drop method, it is challenging to control 3D tissues accurately to the intended shapes because of the inability to impose physical constraints on the 3D tissue.

In recent years, 3D bioprinting methods using material extrusion and stereolithography have attracted attention to fabricating 3D tissues [33,34,35,36]. The application of 3D bioprinting technology has enabled the fabrication of 3D tissues of various shapes. However, in 3D bioprinting, there is a tradeoff between tissue precision and manufacturing costs. In addition, precise control of the fabrication conditions, such as humidity, is required. Therefore, 3D bioprinting is not efficient for creating many samples with high throughput. Furthermore, the method is unsuitable for cell evaluation applications, such as drug discovery technology and regenerative medicine, which require mass production.

**Figure 1:**
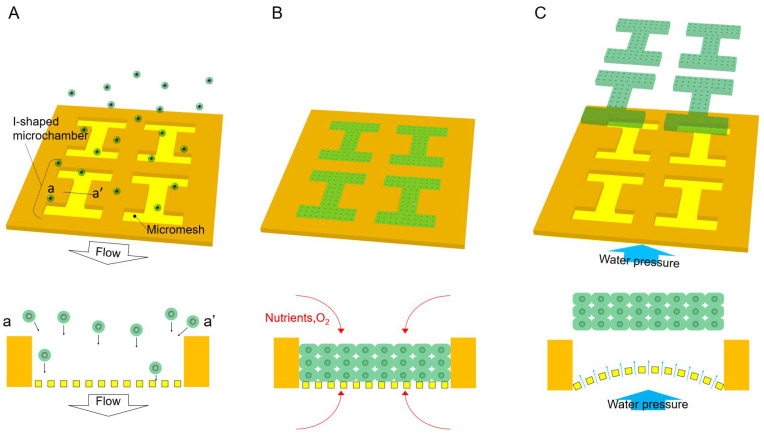
Principle of the proposed fabrication method of shape-controlled 3D tissues using HPPS. (**A**) Spreading cells on the HPPS. Spread cells were guided to the I-shaped microchamber by flow, which was passed through the micromesh. (**B**) By applying oxygen and nutrients from both sides of 3D tissue, cells were cultivated and formed into 3D tissues by adhering to other cells that generate an extracellular matrix. The 3D tissue shape is determined by the I-shaped microchamber. (**C**) After 3D tissues were formed inside the I-shaped microchamber, water pressure was applied from the back of the micromesh. The 3D tissue was released by flow passing through the pore of the micromesh and the expansion and contraction of the micromesh.

In this study, we propose an efficient method for fabricating shape-controlled 3D tissues free of non-natural materials using a high-porosity porous scaffold (HPPS). HPPS consists of a micromesh with high-porosity and an I-shaped microchamber that determines the shape of the 3D tissue (Figure 1A). The micromesh retains the cells inside the microchamber, and the cell adhesion strength is significantly reduced compared with that of the nonporous surface owing to its high porosity. In addition, the micromesh allows the supply of nutrients and oxygen to the 3D tissue from both the upper and lower sides during cultivation (Figure 1B). Thus, it enables the cultivation of thicker 3D tissues than conventional culture dishes. After the cells spread in the I-shaped microchamber to form 3D tissues, the water pressure of the dripping phosphate-buffered saline (PBS) droplets is applied from the opposite side of the cell-spread surface of the micromesh using a pipette to release the 3D tissue (Figure 1C). During this release process, owing to the expansion and contraction of the micromesh of HPPS, the adhesion between the micromesh and 3D tissue weakens. Furthermore, water pressure from the penetrating pores causes the 3D tissue to be pushed out and released from the I-shaped HPPS microchamber. Therefore, shape-controlled 3D tissues can be created using only minimal physical forces without using chemical or biochemical materials, such as protein-degrading enzymes.

To create shape-controlled 3D tissues without chemical reactions, the HPPS must have a micromesh with high porosity, which does not allow cell penetration. Furthermore, the HPPS should have an appropriate cell adhesion strength on its surface for cultivation and release. To meet this design requirement, SU-8, an epoxy-based negative-type photoresist, was used as the HPPS material because the adhesion strength between SU-8 and the adhesive cells is lower than that of materials such as polystyrene and glass [37]. The HPPS was fabricated using SU-8, and the precision accuracy and porosity were evaluated. U-87 human glioma cells were spread on the fabricated HPPS. After the cultivation of U-87 cells, the 3D tissue was released, and the conditions of the constituent cells were observed and evaluated. In addition, the effect of this method on cells was investigated by measuring the viability of the released cells, demonstrating the effectiveness and usefulness of this 3D tissue construction and retrieval technique.

## 2. Materials and Methods

### 2.1. Design and Fabrication Method for HPPS

**Figure 2:**
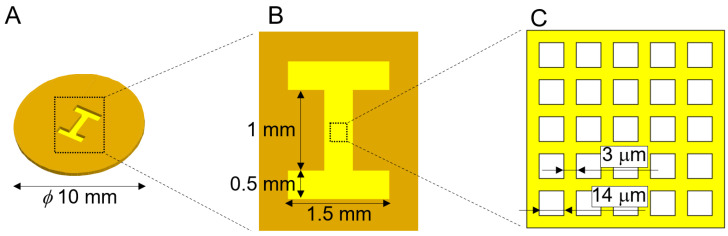
Schematic image of the HPPS with microchamber and micromesh. (**A**) The diameter of the HPPS was 10 mm. (**B**) The microchamber determining the shape of the 3D tissue was I-shaped. The size of the I-shaped microchamber was 2.0 × 1.5 mm, with a minimum width of 0.5 mm. (**C**) Micromesh was on the bottom of the I-shaped microchamber, with a lattice width of 3 μm. The pores on the micromesh were square-shaped with a side length of 14 μm.

The HPPS consists of a micromesh with high porosity and an I-shaped microchamber that determines the shape of the 3D tissue. A schematic of the HPPS design is shown in Figure 2. The HPPS diameter was *ϕ*10 mm (Figure 2A). The size of the I-shaped microchamber was 2.0 × 1.5 mm, with a minimum width of 0.5 mm (Figure 2B). The design values for the high porosity micromesh were a lattice width of 3 μm and a pore size of 14 μm (Figure 2C).

**Figure 3:**
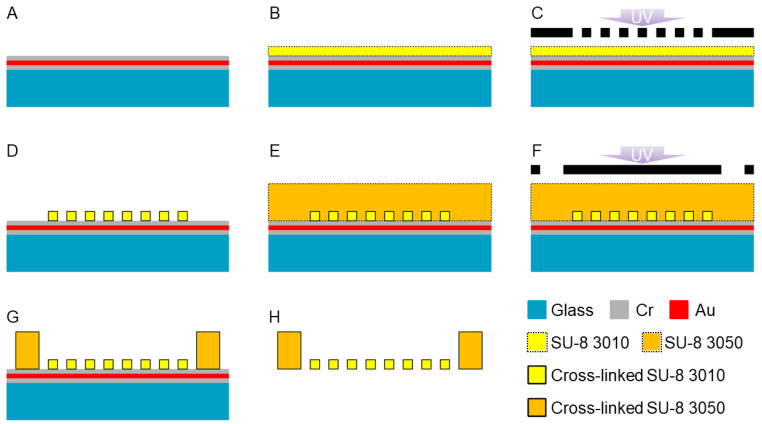
Fabrication process of the HPPS. (**A**) Deposition of Cr/Au/Cr sacrificial layer. (**B**) Deposition of SU-8 3010. (**C**) Exposure of SU-8 3010 layer. (**D**) Development of exposed SU-8 3010 layer. (**E**) Deposition of SU-8 3050. (**F**) Exposure of SU-8 3050 layer. (**G**) Development of SU-8 3050 layer. (**H**) Release of SU-8 structure by etching the sacrificial layer.

The HPPS was fabricated via photolithography using SU-8, an epoxy-based photoresist. A schematic of the HPPS fabrication process is shown in Figure 3. First, the glass substrate (50 × 50 × 1.1 mm, TENPAXGLASS) was washed with piranha solution for 10 min. A Cr/Au/Cr layer with a thickness of 100 nm was deposited via sputtering (10 W-IBS, Hashino-tech, Tokyo, Japan) (Figure 3A). After 10 min in the piranha solution, the oligomeric adhesion promoter (OAP; Tokyo Ohka Kogyo Co., Ltd., Tokyo, Japan) was spin-coated at 4000 rpm for 30 s (MS-B150; Mikasa Co., Ltd., Tokyo, Japan). The oligomeric adhesion promoter layer was baked at 200 °C for 1 min. After cooling to room temperature, SU-8 (SU-8 3010, Nippon Kayaku Co., Ltd., Tokyo, Japan) was spin-coated at 4000 rpm for 30 s. The SU-8 layer was baked at 95 °C for 1 min to volatilize the solvent (Figure 3B). After cooling to room temperature, the SU-8 layer was exposed to light at a wavelength of 365 nm and a dose of 90 mJ/cm^2^ (PEM-800, Union Optical Co., Ltd., Aichi, Japan) (Figure 3C). Furthermore, the SU-8 layer was baked at 95 °C for 1 min. After cooling to room temperature, the SU-8 layer was developed for 20 min using an SU-8 developer (SU-8 developer, Nippon Kayaku Co., Ltd., Tokyo, Japan). After removing the SU-8 developer, the SU-8 layer was rinsed with 2-propanol (32435-00, Kanto Chemical Co., Inc., Tokyo, Japan) (Figure 3D). After hard baking at 150 °C for 20 min and cooling to room temperature, the SU-8 (SU-8 3050, Nippon Kayaku Co., Ltd., Tokyo, Japan) was spin-coated at 400 rpm for 30 s (MS-B150, Mikasa Co., Ltd., Tokyo, Japan). The SU-8 layer was degassed for 30 min in a vacuum chamber to remove the air bubbles. To volatilize the solvent, the SU-8 layer was baked at 95 °C for 3 h (Figure 3E). After cooling to room temperature, the SU-8 layer was exposed to light at a wavelength of 365 nm and a dose of 300 mJ/cm^2^ (PEM-800, Union Optical Co., Ltd., Aichi, Japan) (Figure 3F). The SU-8 layer was baked at 95 °C for 5 min. After cooling to room temperature, the SU-8 layer was developed for 20 min using an SU-8 developer (SU-8 developer, Nippon Kayaku Co., Ltd., Tokyo, Japan). After removing the SU-8 developer, the SU-8 layer was rinsed with 2-propanol (32435-00, Kanto Chemical Co., Inc., Tokyo, Japan) (Figure 3G). HPPS was released from the glass substrate by etching the Cr layer with a Cr etchant (TK, Hayashi Pure Chemical Ind., Ltd., Osaka, Japan). After the release, the HPPS was washed with DI water. (Figure 3H). After drying, the HPPS was bonded to a hollow cylinder with an inner diameter of *ϕ*8 mm, an outer diameter of *ϕ*10 mm, and a height of 5 mm using polydimethylsiloxane (PDMS; SILPOT 184, Dow Corning Toray Co., Ltd., Tokyo, Japan) to form a chamber. An I-shaped microchamber structure without a micromesh, which was the reference structure, was fabricated by applying the process shown in Figure 3C using a photo mask without the pattern of the micromesh structure. The fabricated structures were observed using a scanning electron microscope (SEM; JSM-6060-EDS; JEOL Ltd., Tokyo, Japan).

### 2.2. Cell Culture

U-87-green fluorescent protein (GFP) cells were used as culture cell lines. U-87 cells are human brain tumor cancer cells that express GFP. The medium was prepared by mixing Dulbecco’s modified eagle’s medium (DMEM, 12800-017, Thermo Fisher Scientific Inc., Waltham, MA, USA) with 10 *v*/*v*% fetal bovine serum (FBS, Merck KGaA, Darmstadt, Germany), 1 *v*/*v*% penicillin (P4333, Merck KGaA, Darmstadt, Germany), 2.5 *v*/*v*% HEPES buffer (1M, GB10, FUJIFILM Wako Pure Chemical Corporation, Osaka, Japan), 3.7 g Sodium hydrogen carbonate (191-01305, FUJIFILM Wako Pure Chemical Corporation, Osaka, Japan), 0.1 *v*/*v*% Amphotericin B (15290018, Thermo Fisher Scientific Inc., Waltham, MA, USA), and 0.4% G418 (10131035, Thermo Fisher Scientific Inc., Waltham, MA, USA). U-87 cells were cultured in a 5% CO_2_ atmosphere at 37 °C. Phosphate-buffered saline (PBS, T900, Takara Bio Inc., Shiga, Japan) was used as the cell-washing solution. A 0.11% Trypsin/1 mM EDTA solution (25200056, Thermo Fisher Scientific Inc., Waltham, MA, USA) was used to remove the cells adhered to the bottom of the cell culture dish, which had a diameter of *ϕ*90 mm (S90-NC18; Fine Plus International Ltd., Kyoto, Japan).

### 2.3. Tissue Cultivation and Releasing

**Figure 4:**
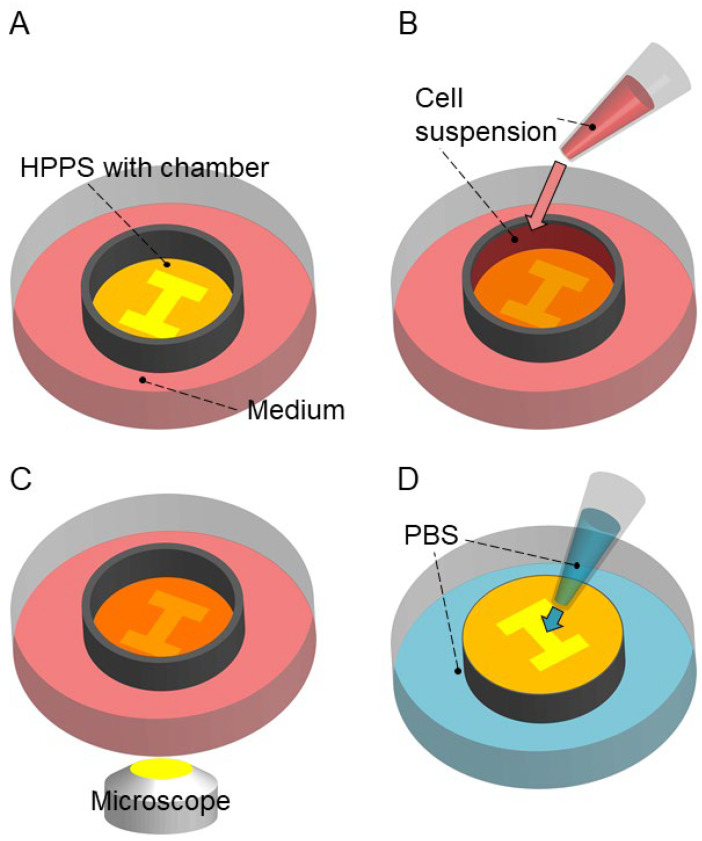
Experimental process of 3D tissue culturing and releasing. (**A**) Setting the HPPS with chamber into the medium in a Petri dish. (**B**) Adding cell suspension into the chamber. (**C**) Culturing U-87 cells on the HPPS. (**D**) Reversing and setting the HPPS with chamber into the Petri dish with PBS. PBS was dripped to the back of the HPPS using a pipette, and the 3D tissue was released into the Petri dish.

Shape-controlled 3D tissue was released without using specific chemicals such as protein-degrading enzymes. The experimental process is illustrated in Figure 4. First, we sterilized the HPPS using 70% ethanol and washed it using PBS three times. By this process, the microchamber of HPPS was fulfilled by PBS even though the SU-8 surface was hydrophobic. Then, we placed the HPPS in a Petri dish and filled the surrounding area with 10 mL of the medium (Figure 4A). Next, we added 200 μL of cell suspension with a concentration of 1.0 × 10^7^ cells/mL into the chamber (Figure 4B). The HPPS with the Petri dish was then placed in an incubator and incubated for 48 h under conditions of 37 °C and 5% CO_2_. After 48 h of incubation, the U-87 cells were observed in HPPS using an inverted fluorescence microscope (IX-73; Evident, Tokyo, Japan). The exposure time was fixed at 0.5 ms (Figure 4C). After removing the HPPS from the Petri dish, it was inverted and placed in another Petri dish filled with PBS. Water pressure was applied to release the 3D tissue in the I-shaped microchamber by dropping PBS onto the rear side of the HPPS micromesh (Figure 4D). Bright-field and fluorescence images were captured after releasing the 3D tissue. To evaluate the viability of the U-87 cells released from HPPS, a cell suspension containing U-87 cells released from HPPS was stained with trypan blue (15250-061, Thermo Fisher Scientific Inc., Waltham, MA, USA) in a 1:1 volume ratio. The percentage of dead cells was calculated using a cell concentration counting chip (DHC-B02N; NanoEnTek Inc., Seoul, Republic of Korea). To observe the U-87 cells remaining on the HPPS surface, the surface after 3D tissue release was observed using a VHX-5000 microscope (Keyence Co., Ltd., Osaka, Japan). Furthermore, to observe the morphological changes in the 3D tissues released from HPPS, the PBS surrounding the released 3D tissues from HPPS was exchanged with the culture medium and incubated for 24 h. After incubation, 3D tissues released from the HPPS were observed using an inverted fluorescence microscope (IX-73; Evident, Tokyo, Japan). The same experiments were conducted using an I-shaped microchamber without a micromesh.

### 2.4. Microscopy and Image Analysis

Fluorescence images of U-87 cells before and after release from the HPPS and U-87 cells 24 h after release were observed using an inverted fluorescence microscope (IX-73, Evident, Tokyo, Japan), CCD camera (DP80, Evident, Tokyo, Japan), and fluorescence filter (U-FBNA, Evident, Tokyo, Japan). Bright-field images of U-87 cells released from HPPS and cultured for 24 h after release were obtained using an inverted fluorescence microscope (CKX41; Evident, Tokyo, Japan) and a CCD camera (DP26; Evident, Tokyo, Japan). The fluorescence intensity of the images was analyzed using the public domain image processing and analysis program ImageJ for Microscopy (NIH).

## 3. Results and Discussion

### 3.1. Fabrication of HPPS

**Figure 5:**
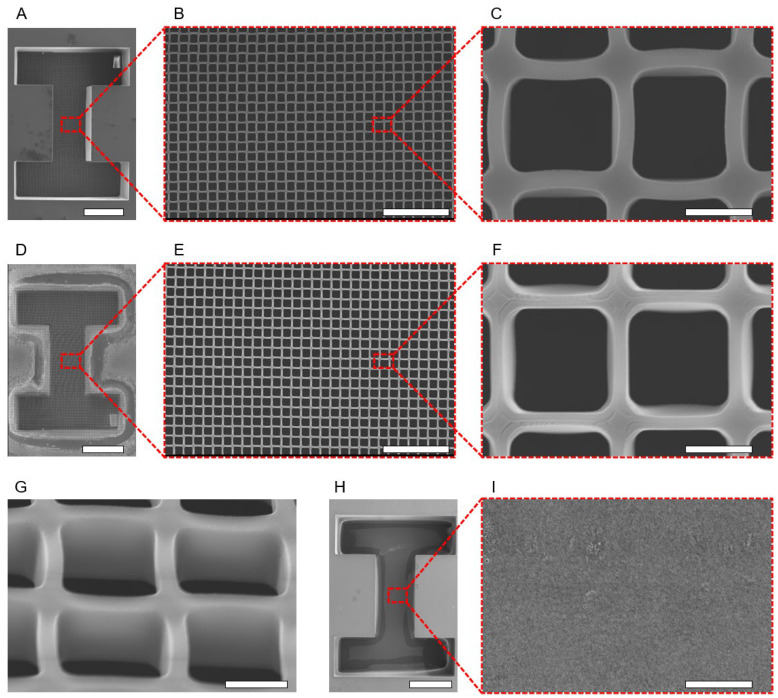
SEM images of HPPS and I-shaped microchamber structure without micromesh. (**A**) SEM image of the top of the HPPS. Scale bar indicates 500 μm. (**B**,**C**) Magnification SEM images of the top of micromesh. Scale bar indicates 100 μm and 10 μm, respectively. (**D**) SEM image of HPPS of the back. Scale bar indicates 500 μm. (**E**,**F**) Magnification SEM images of the back of micromesh. Scale bar indicates 100 μm and 10 μm, respectively. (**G**) Magnification SEM image of micromesh by observing a 45° angle. Scale bar indicates 10 μm. (**H**) SEM image of the top of the I-shaped microchamber without the micromesh. Scale bar indicates 500 μm. (**I**) Magnification SEM image of the bottom surface of an I-shaped microchamber without the micromesh. Scale bar indicates 10 μm.

HPPS was fabricated on a sacrificial layer and released by etching the sacrificial layer. An SEM image of the fabricated HPPS is shown in Figure 5. When the HPPS was observed from the top (cell-seeding side), an I-shaped microchamber that determined the shape of the 3D tissue was observed (Figure 5A). A micromesh was observed on the bottom surface of the I-shaped microchamber (Figure 5B,C). A micromesh was also observed on the rear side of the I-shaped microchamber (Figure 5D–F). Furthermore, the pores on the micromesh were confirmed to have penetrated under observation at a 45° inclination (Figure 5G). As a reference, no micromesh or pores were observed on the bottom surface of the I-shaped microchamber of the HPPS fabricated using the photomask without a micromesh pattern (Figure 5H,I). The sizes of the fabricated HPPS were measured using image analysis, and the measurement results are listed in Table 1. The thicknesses of the micromesh and I-shaped microchamber were 9.96 ± 0.92 μm and 555.26 ± 11.17 μm, respectively. Because the thickness of the 3D tissue was controlled by the I-shaped microchamber, a 3D tissue that was thicker than the thickness of the I-shaped microchamber could not be constructed using the proposed method. Theoretically, in a 3D tissue that has no actual capillaries inside, apoptosis occurs from the inside when the tissue thickness exceeds 200 μm. Therefore, the maximum achievable thickness for 3D tissue without capillaries is approximately 200 μm, and no higher thickness is required [38]. The thickness of the I-shaped microchamber fabricated in this process, 555.26 ± 11.17 μm, is sufficient for creating 3D tissue with a thickness under 200 μm.

The pore width of the micromesh on the top (cell-seeding side) was 14.87 ± 1.83 μm, whereas, on the back, it was 15.51 ± 0.75 μm. The lattice width was 2.24 ± 0.10 μm on the top (cell-seeding side) and 1.72 ± 0.04 μm on the back. The lattice width of the micromesh and the pore size were smaller (25.3–42.7%) and larger (6.2–10.8%) than the designed size. In addition, the lattice width on the top (cell-seeding side) was larger than that on the back. Because of the diffraction of the exposed light and diffusion of acid during the photolithography process, the lattice width on the top (cell-seeding side) was larger than on the back [39]. However, a smaller lattice width is desired to minimize the contact area between the 3D tissue and the micromesh and to release the 3D tissue easily. As a result, the exposure dose of 90 mJ/cm^2^ used for micromesh fabrication was significantly lower than the recommended exposure dose for SU-8 layers with a thickness of approximately 10 μm. Therefore, the fabricated lattice width (1.72–2.24 μm) was smaller compared to the designed size (3.0 μm) (Table 1). In this study, it was important to balance the porosity and strength of the micromesh to reduce the adhesion strength between the cells and the micromesh without breaking it. The micromesh did not break when the water pressure of the dripping PBS droplets was applied. Because the fabricated high-porosity micromesh was sufficiently robust to withstand the water pressure of dripping PBS droplets, it was appropriate for the fabrication of the HPPS, even though the exposure dose of 90 mJ/cm^2^ was lower than the recommended exposure dose. Since the U-87 cells the size of which was from 20 to 30 μm were not passing through the pore of micromesh, the pore size (14.87 μm) of the fabricated micromesh was sufficient to hold the U-87 cells inside the I-shaped microchamber. In addition, since the minimum manufacturable size of the SU-8 is approximately 0.5–1 μm, it is possible to fabricate any size of micromesh suitable for any type of cells by changing the size of micromesh patterns under different conditions of the photomask and the exposure dose.

The porosity of the micromesh was 69.06 ± 3.30% (Figure 5C). Previous studies have utilized micromeshes with through-holes as scaffolds for culturing 3D tissues and creating a mimic of barrier tissues [40,41]. In these studies, a micromesh with high porosity was required because porosity is correlated with the exposure of cells to metabolic substances, stimulants, and oxygen. Kim et al. fabricated a porous membrane with 40% porosity as a scaffold for cell culture [42]. The micromesh fabricated in this study had a higher porosity than that in previous studies. This enabled a more efficient supply of nutrients and oxygen to the 3D tissue. Furthermore, it reduced the adhesive strength between the 3D tissue and micromesh because of the inverse relationship between porosity and adhesive surface area. Therefore, the high-porosity micromesh proposed and fabricated in this study will be useful for creating and facilitating the release of 3D tissues.

In this study, SU-8 was used as the material for the HPPS, considering the processing accuracy. SU-8 is an epoxy-based photoresist commonly used for fabricating micro electro mechanical systems (MEMS) and scaffold structures for biological experiments [37,43,44,45]. Owing to its high fabrication accuracy at the microscale, the proposed method allows the fabrication of suitably sized structures for each type of cell by changing the design of the HPPS. Therefore, the fabrication method for shape-controlled 3D tissues using the HPPS is flexible and scalable. It is suitable for various applications, including regenerative medicine and 3D biological robotics, which require many 3D tissues of various shapes. In contrast, a structure for controlling the shape of 3D tissue, such as an I-shaped microchamber, can be created on a single plane using the same microfabrication process as in the semiconductor fabrication process, which is suitable for mass production. Thus, the productivity of 3D tissue can be enhanced dramatically without changing the fabrication process of the HPPS or the process of creating the 3D tissue. In addition, the fact that the processing time does not depend on the number of 3D tissues created is an advantage of the proposed method over other 3D bioprinting technologies. Therefore, it was expected that the proposed method would be useful for the reasonable manufacturing of 3D tissue compared to 3D bioprinters.

### 3.2. Tissue Cultivation and Releasing

**Figure 6:**
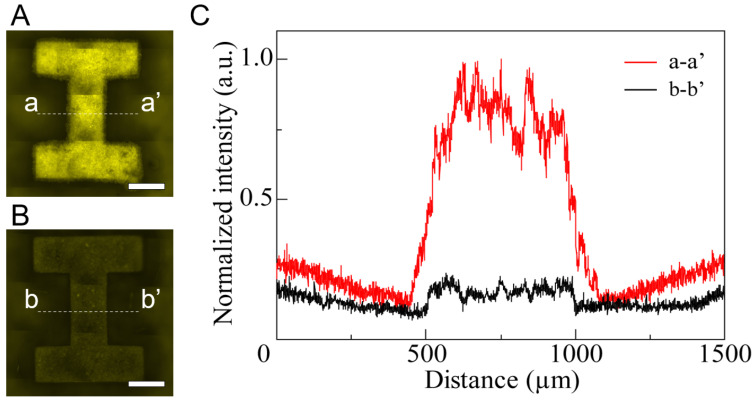
Fluorescence images of U-87 cells for 48 h cultivation after spreading. (**A**) U-87 cells on HPPS. Scale bar indicates 500 μm. (**B**) U-87 cells on an I-shaped microchamber without a micromesh. Scale bar indicates 500 μm. (**C**) Normalized fluorescence intensity in cross-section in (**A**,**B**) (the dot-dashed lines: a-a’ and b-b’).

U-87 cells were spread on HPPS and I-shaped microchamber structures without a micromesh. Fluorescence images were captured using an inverted fluorescence microscope (IX-73, Evident, Tokyo, Japan), a CCD camera (DP80, Evident, Tokyo, Japan), and a fluorescence filter (U-FBNA, Evident, Tokyo, Japan). Fluorescence images of the U-87 cells in the HPPS and I-shaped microchamber structures without a micromesh after 48 h of cultivation are shown in Figure 6A,B, respectively. The exposure time for the fluorescence microscopy observation was 0.5 ms. The fluorescence of U-87 cells was observed in both structures. The fluorescence intensities of the cross-sectional parts (dot-dashed lines: a-a’, b-b’) in the I-shaped microchambers are shown in Figure 6C. The fluorescence intensity in the I-shaped microchamber was higher than that in other areas. By contrast, the fluorescence intensity of the I-shaped microchamber with the micromesh was approximately five times higher than that of the I-shaped microchamber without the micromesh. Although the initial cell number was the same in both devices, a higher number of U-87 cells were viable in the I-shaped chamber of the HPPS after 48 h of cultivation. Thus, the viability of U-87 cells inside the I-shaped microchamber was maintained by efficiently supplying nutrients and oxygen through the pores of the high-porosity micromeshes. Previous research has shown that porous materials and membranes that allow the permeation of nutrients and oxygen support efficient culture, and the results of this research are similar to those of previous research [46,47].

**Figure 7:**
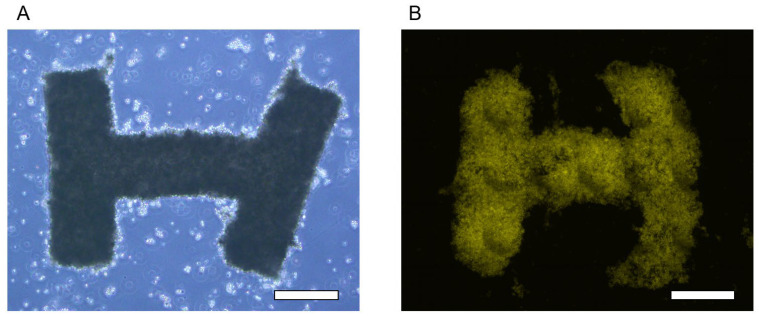
The microscopic images of 3D tissue released from the HPPS. (**A**) The bright-field image of the 3D tissue. (**B**) The fluorescence image of the 3D tissue. Scale bars indicate 500 μm.

After 48 h of cultivation, U-87 cells were released from the I-shaped microchambers by applying water pressure and dripping PBS droplets using a pipette. When the U-87 cells inside the I-shaped microchamber without the micromesh were released, the U-87 cells were released in pieces (without forming the 3D shape) because they did not attach and were not 3D shaped. In contrast, the 3D tissue was released in an I-shape from the HPPS I-shaped microchamber. The released 3D tissue maintained its I-shape for 1–2 h after release (Figure 7A). Fluorescence from the 3D tissue was also observed in the culture dish using a fluorescence microscope (Figure 7B). Thus, it was indicated that the I-shaped microchamber could provide sufficient nutrients and oxygen to the cells for cell adhesion and the formation of 3D tissue. In the proposed method using HPPS, the temporary adhesion of U-87 cells to the micromesh and inner walls of the I-shaped microchamber was used to control the shape of the 3D tissue. However, the adhesion strength of the 3D tissue to the I-shaped microchamber when the 3D tissue is released must be sufficiently weak. Because the released 3D tissue maintained an I-shape, the adhesion strength between the SU-8 micromesh/inner wall and U-87 cells was appropriate for controlling the cell shape and facilitating the release process.

**Figure 8:**
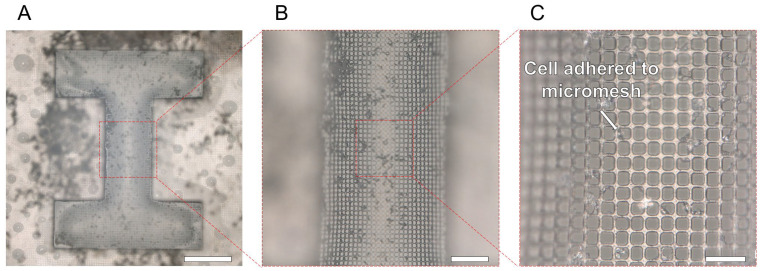
Photograph of the HPPS after the 3D tissue was released. (**A**) Photograph of I-shaped microchamber on HPPS. Scale bar indicates 500 μm. (**B**,**C**) Enlarged views of the micromesh and remaining cells on the HPPS. Scale bars indicate 150 μm and 50 μm.

The percentage of non-living cells immediately after release measured using trypan blue was 2.44 ± 0.70% for HPPS and 30.42 ± 18.93% for I-shaped chambers without micromesh. Therefore, more than 90% of the U-87 cells in the HPPS survived. The efficacy of the HPPS micromesh in providing sufficient nutrients and oxygen to the cells was demonstrated. The surface of the micromesh after the 3D tissue release is shown in Figure 8. Since the water pressure generated by only dropping the PBS water droplet was very low, there was no damage to the HPPS micromesh. Most U-87 cells spread on the I-shaped microchamber and were released as 3D tissues with an I-shape. The 3D tissue on the micromesh is released easily for three reasons. First, the cell adhesion area on the micromesh was approximately 70% smaller than that on the non-micromesh areas. Second, water pressure was generated from the pores when the droplets were dripping, and a small physical force was applied to the U-87 cells. Third, because the thickness of the micromesh was less than 10 μm, it was easily deformed (stretched) when the water pressure of the dripping PBS droplets was applied, thereby reducing the adhesion strength to the cells. For these reasons, using a thin micromesh with high porosity allows for the simple and harmless production of 3D tissue.

**Figure 9:**
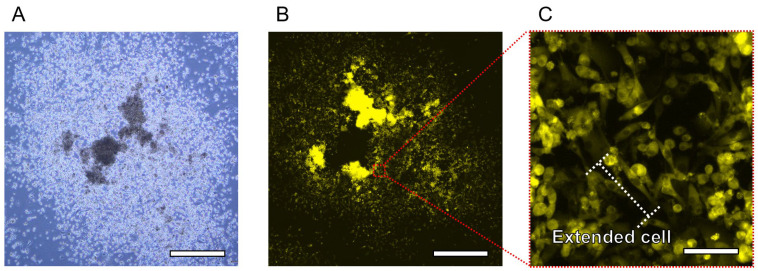
Microscopic images of the 3D tissue during 24 h cultivation after the releasing process. (**A**) The bright-field image of the deconstructed 3D tissue. Scale bar indicates 1 mm. (**B**) The fluorescence image of the deconstructed 3D tissue. Scale bar indicates 1 mm. (**C**) Enlarged view of the fluorescence image of the deconstructed 3D tissue. Scale bar indicates 30 μm.

The released 3D tissue was cultured for 24 h in a Petri dish. The bright-field and fluorescence images of the released 3D tissue after 24 h are shown in Figure 9. Bright-field and fluorescence images of the deconstructed 3D tissue are shown in Figure 9A,B, respectively. The 3D tissue started disassembling 1 h after the release. The 3D tissue lost its shape 24 h after release. An enlarged view of the deconstructed 3D tissue is shown in Figure 9C. Most cells were attached to each other or to the bottom of the Petri dish and extended. Because the cells that were part of the released 3D tissue adhered and extended to the bottom of the Petri dish, the cells that had composed the 3D tissue were not severely damaged during the release. However, because the 3D tissue produced by this method did not contain any non-natural material, it could not maintain its shape for a long time after being released from the I-shaped microchamber of the HPPS. In future work, by mixing natural materials such as collagen, it will be possible to improve the bonding strength of the tissue and maintain the shape of the three-dimensional tissue for a longer time than in this experiment.

In this study, we created shape-controlled 3D tissues free of non-natural materials. Even though it was difficult to maintain its shape long-term without any materials to support it, the 3D tissue maintained its intended shape for 1 h, and the cells consisting of the 3D tissue were not damaged. This characteristic of the created 3D tissue is useful in regenerative medicine, which requires 3D tissue that does not contain non-natural materials, 3D tissue with a designed size and shape, and biological robotics, which uses the self-assembly of 3D tissues. In addition, because the HPPS can be fabricated using photolithography, which is part of the manufacturing process for semiconductors suitable for fabrication precision, it is easy to change the design of the HPPS for each type of cell. In addition, the proposed method has potential applications in the biological industry that require mass production because microchambers for determining the shape of 3D tissue can be created multiply, which is like semiconductors, on a flat surface without changing and adding extra fabrication processes and costs. Although the 3D tissue created in this study cannot withstand long-term culture, if we can create stronger cell-to-cell interactions within the 3D tissue using natural materials (collagen, fibronectin, etc.) in the future, long-term evaluation also becomes possible. Therefore, the proposed method has a high possibility of contributing to the biological industry, such as drug development and regenerative medicine, by carrying out more experiments for evaluating the fabricated 3D tissues’ function, such as the metabolic function, etc., as future work.

## 4. Conclusions

In this study, we proposed a novel fabrication method for shape-controlled 3D tissues without non-natural materials using HPPS. Shape-controlled 3D tissue was created only by applying water pressure without using chemicals to deconstruct 3D tissue, such as proteolytic enzymes. The 3D tissue had a designed I-shape, and the viability of the cells in the 3D tissue was over 90%. It is easy to change the design at the microscale and is appropriate for large-scale manufacturing because the HPPS was fabricated using common photolithography in semiconductor manufacturing. Therefore, the proposed method for creating shape-controlled 3D tissues free of non-natural materials without specific chemicals is easy to apply for various biological applications. Furthermore, the technique causes minimal cell damage and is suitable for mass production, which the biological industry requires.

## Figures and Tables

**Table 1:** Designed and fabricated size of the HPPS.

	Designed (μm)	Measured (μm)
Pore (Top)	14	14.87 ± 1.83
Pore (Back)		15.51 ± 0.75
Lattice (Top)	3	2.24 ± 0.10
Lattice (Back)		1.72 ± 0.04
Thickness (Micromesh)	10	9.96 ± 0.92
Thickness (Microchamber)	>200	555.26 ± 11.17

## Data Availability

Data are contained within the article.
